# Characterization of the complete chloroplast genome of *Magnolia wilsonii* (Magnoliaceae)

**DOI:** 10.1080/23802359.2019.1678425

**Published:** 2019-10-18

**Authors:** Li-Zhen Ling, Shu-Dong Zhang

**Affiliations:** School of Biological Sciences and Technology, Liupanshui Normal University, Liupanshui, China

**Keywords:** Chloroplast genome, phylogenetic analysis, *Magnolia wilsonii*, Magnoliaceae

## Abstract

The complete chloroplast (cp) genome of *Magnolia wilsonii*, an endangered and endemic species in China, was first reported in this study. The *M. wilsonii* cp genome was 157,434 bp in length, including two inverted repeat (IR) regions of 26,868 bp, a large single copy (LSC) region of 85,387 bp, and a small single copy (SSC) region of 18,311 bp. A total of 113 unique genes, including 79 protein-coding genes, 4 ribosomal RNA genes, and 30 transfer RNA genes, were annotated. The overall GC content was 38.8%. Phylogenetic analysis of 34 representative cp genomes within the genus *Magnolia* suggests that *M. wilsonii* was sister to *M. sieboldii*.

*Magnolia wilsonii* (Finet & Gagnepain) Rehder is an evergreen tree belonging to sect. *Oyama* Nakai in the family of Magnoliaceae (Xia et al. 2008). It is endemic to China and distributes in montane forest and thicket between 1,900 and 3,300 m elevation in western Sichuan, northern Yunnan and western Guizhou (Xia et al. 2008; Workshop 2015). The bark of *M. wilsonii* has been used to clear head and chest congestion and for intestinal relief for centuries in China (Liu 2004). *Magnolia wilsonii* is usually considered as the excellent ornamental tree species due to the large, white and fragrant blooms (Liu 2004). At now, *M. wilsonii* is classified as the national second-grade protection of China due to habitat loss and fragmentation and the decline in population size (Workshop 2015). To promote the conservation of this species, we sequenced and characterised the complete chloroplast (cp) genome of *M. wilsonii* using Illumina sequencing technology.

In this study, a new discovery of distribution of *M. wilsonii* was recorded in Motuo county, Xizang (Tibet, N29°41′45″, E95°32′35″, 2,700 m), China. Specimens (13CS6544) were deposited at Herbarium, Kunming Institute of Botany, CAS (KUN). The total DNA was extracted from gel-dried leaf described as the previous study (Ling and Zhang 2019). The amount of 5 µg of total DNA was used for the library construction and the subsequent high-throughput sequencing on Illumina Hiseq 2500 Platform. About 2 Gb raw data were used to *de novo* assemble the complete cp genome using the GetOrganelle pipeline (https://github.com/Kinggerm/GetOrganelle). All genes encoding proteins, transfer RNAs (tRNAs), and ribosomal RNAs (rRNAs) were automatically annotated using Dual Organellar Genome Annotator (DOGMA) with manual adjustments (Wyman et al. 2004). The complete cp genome sequence of *M. wilsonii* was deposited in GenBank under the accession number MN326013.

The cp genome of *M. wilsonii* was 157,434 bp in length, including a pair of inverted repeat (IR) regions of 26,868 bp each, a large single-copy region (LSC) of 85,387 bp, and a small single-copy region (SSC) of 18,311 bp. The cp genome of *M. wilsonii* showed a GC content of 38.8% and contained a total of unique 113 genes, consisting of 79 protein-encoding genes, 30 tRNA genes, and 4 rRNA genes. Of them, 15 genes (*atpF*, *ndhA*, *ndhB*, *petB*, *petD*, *rpl16*, *rpl2*, *rpoC1*, *rps16, trnA-UGC*, *trnG-GCC*, *trnI-GAU*, *trnK-UUU*, *trnL-UAA,* and *trnV-UAC*) were composed of a single intron. Three genes (*clpP, rps12* and *ycf3*) contained two introns.

Phylogenomic analysis was performed with the maximum likelihood (ML) and Bayesian inference (BI) methods (Ronquist et al. 2012; Stamatakis 2014). Two species (*Liriodendron chinense* and *L. tulipifera*) were used as the outgroups. The complete cp genomes of *M. wilsonii* and other 33 species of the genus of *Magnolia* were used for phylogenetic analyses (Figure 1). All the cp genome sequences were aligned using MAFFT (Katoh and Standley 2013). The ML and BI analyses generated the same tree topology (Figure 1)*. Magnolia wilsonii* is sister to *M. sieboldii*, another species of sect. *Oyama* of the genus of *Magnolia*.

**Figure 1.**
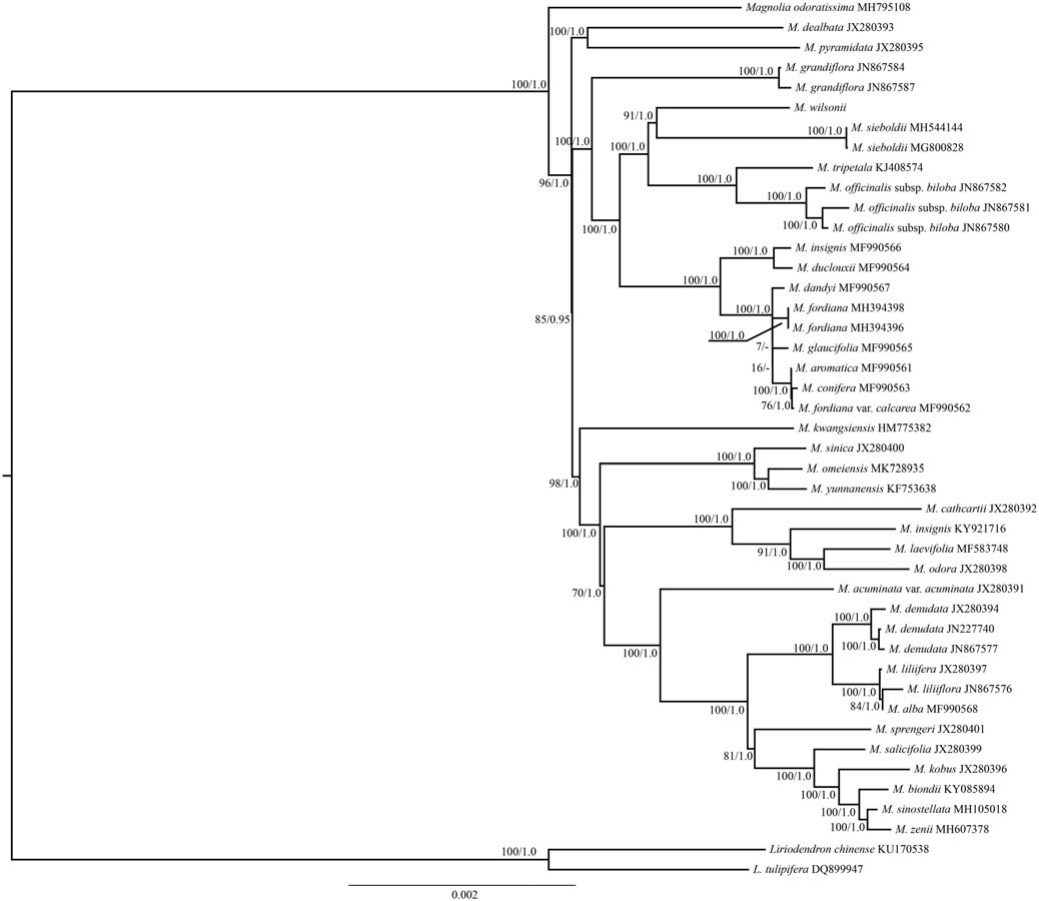
The maximum likelihood (ML) tree of *Magnolia* inferred from the complete chloroplast genome sequences. Numbers at nodes correspond to ML bootstrap percentages (1000 replicates) and Bayesian inference (BI) posterior probabilities.
